# Supplementary material for: Adaptive design methods in dialysis clinical trials: a systematic review protocol
Source: BMJ Open. 2020 Aug 27;10(8):e036755. doi: 10.1136/bmjopen-2019-036755 (PMC7454175; doi:10.1136/bmjopen-2019-036755)
Supplement: Author's manuscript [file bmjopen-2019-036755.draft_revisions.pdf]

BMJ Open is committed to open peer review. As part of this commitment we make the peer review history of every article we publish publicly available.

When an article is published we post the peer reviewers' comments and the authors' responses online. We also post the versions of the paper that were used during peer review. These are the versions that the peer review comments apply to.

The versions of the paper that follow are the versions that were submitted during the peer review process. They are not the versions of record or the final published versions. They should not be cited or distributed as the published version of this manuscript.

BMJ Open is an open access journal and the full, final, typeset and author-corrected version of record of the manuscript is available on our site with no access controls, subscription charges or pay-per-view fees (http://bmjopen.bmj.com).

If you have any questions on BMJ Open's open peer review process please email info.bmjopen@bmj.com

BMJ Open

Adaptive design methods in dialysis clinical trials– a systematic review protocol

Journal:	*BMJ Open*
Manuscript ID	bmjopen-2019-036755
Article Type:	Protocol
Date Submitted by the Author:	02-Jan-2020
Complete List of Authors:	Judge, Conor; National University of Ireland Galway, Department of Medicine<br>Murphy, Robert P; National University of Ireland Galway, ; University Hospital Galway,<br>Cormican, Sarah; National University of Ireland Galway, Department of Medicine<br>Smyth, Andrew; National University of Ireland Galway, Department of Medicine<br>O'Halloran, Martin; National University of Ireland Galway, Department of Medicine<br>O'Donnell, Martin; National University of Ireland Galway, Department of Medicine
Keywords:	Dialysis < NEPHROLOGY, End stage renal failure < NEPHROLOGY, Clinical trials < THERAPEUTICS

SCHOLARONE™
Manuscripts

BMJ

I, the Submitting Author has the right to grant and does grant on behalf of all authors of the Work (as defined in the below author licence), an exclusive licence and/or a non-exclusive licence for contributions from authors who are: i) UK Crown employees; ii) where BMJ has agreed a CC-BY licence shall apply, and/or iii) in accordance with the terms applicable for US Federal Government officers or employees acting as part of their official duties; on a worldwide, perpetual, irrevocable, royalty-free basis to BMJ Publishing Group Ltd ("BMJ") its licensees and where the relevant Journal is co-owned by BMJ to the co-owners of the Journal, to publish the Work in this journal and any other BMJ products and to exploit all rights, as set out in our licence.

The Submitting Author accepts and understands that any supply made under these terms is made by BMJ to the Submitting Author unless you are acting as an employee on behalf of your employer or a postgraduate student of an affiliated institution which is paying any applicable article publishing charge ("APC") for Open Access articles. Where the Submitting Author wishes to make the Work available on an Open Access basis (and intends to pay the relevant APC), the terms of reuse of such Open Access shall be governed by a Creative Commons licence – details of these licences and which Creative Commons licence will apply to this Work are set out in our licence referred to above.

Other than as permitted in any relevant BMJ Author's Self Archiving Policies, I confirm this Work has not been accepted for publication elsewhere, is not being considered for publication elsewhere and does not duplicate material already published. I confirm all authors consent to publication of this Work and authorise the granting of this licence.

For peer review only

Title

Adaptive design methods in dialysis clinical trials– a systematic review protocol

Authors

Conor Judge, M.B., BEng. (1) (2) (3)

Robert Murphy, M.B. (1)

Sarah Cormican, M.B. (1)

Andrew Smyth, M.B., Ph.D. (1)

Martin O Halloran, BEng., Ph.D. (2)

Martin J. O'Donnell, M.B., Ph.D. (1)

Author affiliations

(1) HRB-Clinical Research Facility, NUI Galway, Galway, Ireland

(2) Translational Medical Device Lab, NUI Galway, Galway, Ireland

(3) Wellcome Trust – HRB, Irish Clinical Academic Training

Corresponding Author

Dr Conor Judge, HRB-Clinical Research Facility, National University of Ireland Galway, Newcastle Road, Galway, Ireland, H91YR71

For peer review only

Word Count

1449

For peer review only

Abstract

Introduction

Adaptive design methods are a potential solution to improve efficiency of clinical trials but their uptake in dialysis is unknown. We aim to investigate the use of adaptive design methods in dialysis clinical trials and to cultivate further adoption of adaptive trials by the nephrology community.

Methods and analysis

We will adhere to the Preferred Reporting Items for Systematic Review and Meta-Analysis Protocols (PRISMA-P) and by the Cochrane Collaboration Handbook. We will perform a literature search though MEDLINE (Pubmed), EMBASE and CENTRAL, a detailed data extraction of trial characteristics and a narrative synthesis of the data. There will be no language restrictions. We will estimate the number of adaptive clinical trials per 1000 registered trials per year in dialysis. Sub-group analysis will be performed by dialysis modality, funder and geographical location.

Ethics and dissemination

Ethical approval will not be required for this study as data will be obtained from publicly available clinical trials. We will disseminate our results in a peer-reviewed publication.

Registration Number

PROSPERO: 163946 (Temporary ID)

Keywords

Dialysis, Adaptive design, Haemodialysis, Peritoneal Dialysis, End Stage Renal Disease

For peer review only

Article Summary

Strengths and Limitations

- This study presents a comprehensive methodology for a systematic review of the current state of adaptive designs (AD) clinical trials in dialysis.
- Two researchers will independently perform the study selection, data extraction and risk of bias assessment.
- Detailed characteristics of trials will be collected and analysed including nature of intervention, funding source, dialysis modality, and the nature of the adaptive design.
- Subgroup analysis will be performed to highlight differences in design characteristics between dialysis modality, temporal trends, types of intervention, funding and geographical location.

For peer review only

Introduction

Background

Randomised controlled trials are the gold standard for confirming efficacy or futility of new therapies (1). Nephrology as a speciality, and especially patients with End Stage Renal Disease (ESRD), has traditionally had a low number of randomised trials compared to similar specialities (2). Many reasons have been cited for this including recruitment difficulty, history of underpowered trials and lack of funding (3,4). Nephrology studies are increasing in number but still lag behind other specialities (5).

There are an estimated 726,331 prevalent cases of ESRD in the United States (6) and globally, in 2010, an estimated 2.3–7.1 million people with end-stage kidney disease died without access to chronic dialysis (7). Projections estimate a 11%–18% increase in the crude incidence rate from 2015 to 2030 (8). People with ESRD represent 1% of the Medicare population but account for 7% of Medicare's expenditures (9). Additionally, people with End Stage Renal Disease experience a greater deal of mortality (10), morbidity and worse quality of life than the general public (11).

Adaptive clinical trials would appear particularly suitable for evaluation of novel interventions in ESRD and according to the Food and Drug Administration could reduce resource requirements, decrease time to study completion and increase the likelihood of study success (12).

Objectives

The systematic review will aim to: 1) summarise the use of adaptive design methods in dialysis clinical trials per year, 2) describe the characteristics of the adaptive designs including dialysis modality, funding, and geographical location, 3) describe the adaptive design characteristics of the trial, 4) estimate the number of adaptive clinical trials per 1000 clinical trials in dialysis and 5) outline trends in all the above.

For peer review only

Methods and analysis

This systematic review has been registered with PROSPERO (registration number: 163946 (Temporary ID)). We will conduct this systematic review according to the Preferred Reporting Items for Systematic review and Meta-analysis Protocols (PRISMA-P) and have completed the PRISMA-P checklist (Supplementary Appendix 1).

Inclusion/exclusion criteria for the selection of studies

Type of study design and participants

Randomised controlled trials of interventions in patients with End Stage Renal Disease or acute kidney injury undergoing renal replacement therapy including haemodialysis, peritoneal dialysis, haemodiafiltration and haemofiltration. We will not limit our population or any specific disease.

Type of intervention

We will not place a restriction on the intervention type and will include trials that study medications during dialysis, medical devices, dialysis parameters etc.

Type of outcome

We will include all outcomes including surrogate markers, patient-centred outcomes and hard clinical outcomes.

Search method for the identification of trials

Electronic search

We will perform electronic searches on MEDLINE (Pubmed), EMBASE and CENTRAL from database inception until 01 January 2020. Zotero will be used as our reference manager and the Revtools

For peer review only

package on R will be used to eliminate duplicate records. The search will be conducted in English. The dialysis search terms were adapted from Beaubien-Souligny et al. 2019 (13) and include dialysis[tiab] OR peritoneal dialysis[tiab] OR hemodialysis[tiab] OR hemodiafiltration[tiab] OR haemodiafiltration[tiab] OR hemofiltration[tiab] OR haemofiltration OR extracorporeal blood cleansing[tiab] OR haemodialysis[tiab] OR Renal Dialysis[mh] OR Renal replacement[tiab] OR end stage kidney[tiab] OR end stage renal[tiab] OR stage 5 kidney[tiab] OR stage 5 renal[tiab]).

The adaptive design search terms were adapted from Bothwell et al., 2018 (14) and include "phase ii/iii"[tiab] OR "treatment switching"[tiab] OR "biomarker adaptive"[tiab] OR "biomarker adaptive design"[tiab] OR "biomarker adjusted"[tiab] OR "adaptive hypothesis"[tiab] OR "adaptive dose-finding"[tiab] OR "pick-thewinner"[tiab] OR "drop-the-loser"[tiab] OR "sample size re-estimation"[tiab] OR "re-estimations"[tiab] OR "adaptive randomization"[tiab] OR "group sequential"[tiab] OR "adaptive seamless"[tiab] OR "adaptive design"[tiab] OR "Interim monitoring"[tiab] OR "Bayesian adaptive"[tiab] OR "Flexible design"[tiab] OR "Adaptive trial"[tiab] OR "play-the-winner"[tiab] OR "adaptive method"[tiab] OR (adaptive[All Fields] AND dose[All Fields] AND adjusting[All Fields]) OR "response adaptive"[All Fields] OR "adaptive allocation"[All Fields] OR "adaptive signature design"[tiab] OR "treatment adaptive"[tiab] OR "covariate adaptive"[tiab] OR "sample size adjustment"[tiab]. We will perform two searches, firstly, we will combine the dialysis and adaptive search terms using a Boolean AND. The specific search will be carried out as described in Table 1. Secondly, we will perform a search with the dialysis search terms without the adaptive search terms for calculation of our total randomised controlled trials in dialysis denominator.

Selection and analysis of trials

We will use the high sensitivity machine learning classifier (RobotSearch) to identify randomised controlled trials from the combined search (dialysis and adaptive design) and from the dialysis search (15). RobotSearch is a machine learning classification algorithm combining an ensemble of

For peer review only

Support Vector Machines (SVM) and Convolutional Neural Networks (CNN) with a reported Area Under the Curve of 0.987 (95% Confidence Interval (CI), 0.984 to 0.989). We will then review the title and abstracts of studies to identify trials with adaptive design methods for inclusion or exclusion. Studies with insufficient information to determine use of adaptive design methods will also be included for full text review. We will then perform full text review to confirm studies that will be included in the final systematic review. This process will be summarised in a PRISMA flowchart. Abstract, title and full text review will be performed by CJ and RM. Disagreements will be resolved by consensus or by a third reviewer (SC), if necessary.

CJ and RM will extract the following information (Adapted from Hatfield et al. 2016) in parallel and record in a custom database (Summarised in Table 2):

- The type of the adaptive design e.g. dose-finding, adaptive hypothesis, group sequential, adaptive randomisation, seamless phase II/III, adaptive treatment-switching, biomarker adaptive, sample size re-estimation.
- Stopping rule (Futility or efficacy).
- Year of completion of study.
- Trial population.
- Duration of time participants were receiving renal replacement therapy (Acute Kidney Injury or End Stage Kidney Disease).
- Intervention.
- Domain of the intervention (Medication, medical device or dialysis parameter).
- Primary outcome measure.
- Type of primary outcome variable (Continuous or dichotomous).
- Nature of primary outcome (Surrogate, patient-centered or hard clinical).
- Dialysis Modality (haemodialysis, peritoneal dialysis, haemodiafiltration or haemofiltration).
- Sample Size.

For peer review only

- The country of the lead investigator.
- The funder of the study (Public or Private).
- Published in journal with Impact Factor >10.

Assessment of the quality of the studies: risk of bias

We will use the Cochrane Risk of Bias Tool (16) to assess methodological quality of eligible trials including random sequence generation, allocation concealment, blinding of participants and health care personnel, blinded outcome assessment, completeness of outcome data, evidence of selective reporting and other biases. Risk of bias assessments will be performed independently by two reviewers (SC, RM), and disagreements were resolved by a third reviewer (CJ). If two of the domains was rated as high, the study was considered at high risk of bias. We will create a risk of bias summary table using Review Manager 5.3 (17).

Data synthesis

A descriptive synthesis of the data will be performed. We will estimate the number of adaptive clinical trials per 1000 clinical trials in dialysis per year by dividing the adaptive clinical trials per year by the total number of randomised controlled trials per year and multiplying by 1000.

Analysis by subgroups

We will report overall outcomes and outcomes by dialysis modality (haemodialysis, peritoneal dialysis, haemodiafiltration and haemofiltration).

Study status

This systematic review will start in December 2019.

For peer review only

Patient and public involvement

There was no formal patient and public involvement in the design of this study.

Ethics and dissemination

Ethical approval was not required for this study. We will publish the results of this systematic review in a peer reviewed journal.

Discussion

The number of clinical trials performed in End Stage Renal Disease is low compared to other medical subspecialties (18). Additionally, the cost of conducting randomised controlled trials are rising (19) and there are reduced funding sources available, especially for nephrology (4). Adaptive clinical trials hold the potential to increase the efficiency and number of randomized controlled trials in End Stage Renal Disease and dialysis.

We expect to provide the following results: firstly, we will report on the proportion of randomised controlled trials in dialysis that include an adaptive design, secondly, we will outline the most popular type of Adaptive design in End Stage Renal Disease trials, thirdly, we will report the main dialysis modalities where adaptive designs are used and possibly highlight underutilisation in additional modalities and finally we will report the geographical locations and funding pattern for trials using adaptive design in dialysis.

Furthermore, this systematic review we increase awareness in the nephrology community of the potential benefit of using adaptive designs in clinical trials. Having clear examples and use cases of successful adaptive design will stimulate further use of these designs.

For peer review only

Limitations

This review will have potential limitations including publication and reporting bias. We will not be able to include studies with unpublished data and we will mis-classify studies that do not have clear reporting of adaptive designs in their methodology.

For peer review only

Contributors

CJ, RB and MOD designed the study. All authors reviewed and approved the final version of the protocol. CJ is the guarantor of the review.

Funding

This work was performed within the Irish Clinical Academic Training (ICAT) Programme, supported by the Wellcome Trust and the Health Research Board (Grant Number 203930/B/16/Z), the Health Service Executive, National Doctors Training and Planning and the Health and Social Care, Research and Development Division, Northern Ireland. The funding source had no role in the study design, analysis or writing of report.

Competing interests

None Declared.

Patient consent for publication

Not required.

Provenance and peer review

For peer review only

References

1. Bothwell LE, Greene JA, Podolsky SH, Jones DS. Assessing the Gold Standard — Lessons from the History of RCTs. N Engl J Med. 2016 Jun 2;374(22):2175–81.

2. Kovesdy CP. Clinical trials in end-stage renal disease—priorities and challenges. Nephrol Dial Transplant. 2019 Jul 1;34(7):1084–9.

3. Baigent C, Herrington WG, Coresh J, Landray MJ, Levin A, Perkovic V, et al. Challenges in conducting clinical trials in nephrology: conclusions from a Kidney Disease—Improving Global Outcomes (KDIGO) Controversies Conference. Kidney Int. 2017 Aug 1;92(2):297–305.

4. Bryan L, Ibrahim T, Zent R, Fischer MJ. The Kidney Research Predicament. J Am Soc Nephrol JASN. 2014 May;25(5):898–903.

5. Chatzimanouil MKT, Wilkens L, Anders H-J. Quantity and Reporting Quality of Kidney Research. J Am Soc Nephrol. 2019 Jan 1;30(1):13–22.

6. UNITED STATES RENAL DATA SYSTEM: Chapter 1: Incidence, Prevalence, Patient Characteristics, and Treatment Modalities 2018. [Internet]. [cited 2019 Nov 18]. Available from: http://www.usrds.org

7. Liyanage T, Ninomiya T, Jha V, Neal B, Patrice HM, Okpechi I, et al. Worldwide access to treatment for end-stage kidney disease: a systematic review. The Lancet. 2015 May;385(9981):1975–82.

8. McCullough KP, Morgenstern H, Saran R, Herman WH, Robinson BM. Projecting ESRD Incidence and Prevalence in the United States through 2030. J Am Soc Nephrol. 2019 Jan 1;30(1):127–35.

9. UNITED STATES RENAL DATA SYSTEM: Chapter 9: Healthcare Expenditures for Persons with ESRD [Internet]. 2018 [cited 2019 Nov 18]. Available from: http://www.usrds.org

10. Robinson BM, Zhang J, Morgenstern H, Bradbury BD, Ng LJ, McCullough KP, et al. Worldwide, mortality risk is high soon after initiation of hemodialysis. Kidney Int. 2014 Jan 1;85(1):158–65.

11. Wyld M, Morton RL, Hayen A, Howard K, Webster AC. A Systematic Review and Meta-Analysis of Utility-Based Quality of Life in Chronic Kidney Disease Treatments. Turner N, editor. PLoS Med. 2012 Sep 11;9(9):e1001307.

12. Research C for DE and. Adaptive Design Clinical Trials for Drugs and Biologics [Internet]. U.S. Food and Drug Administration. 2019 [cited 2019 Nov 18]. Available from: http://www.fda.gov/regulatory-information/search-fda-guidance-documents/adaptive-design-clinical-trials-drugs-and-biologics

13. Beaubien-Souligny W, Kontar L, Blum D, Bouchard J, Denault AY, Wald R. Meta-analysis of Randomized Controlled Trials Using Tool-Assisted Target Weight Adjustments in Chronic Dialysis Patients. Kidney Int Rep. 2019 Jul;S2468024919314044.

14. Bothwell LE, Avorn J, Khan NF, Kesselheim AS. Adaptive design clinical trials: a review of the literature and ClinicalTrials.gov. BMJ Open. 2018 Feb 1;8(2):e018320.

For peer review only

15. Marshall IJ, Noel-Storr A, Kuiper J, Thomas J, Wallace BC. Machine learning for identifying Randomized Controlled Trials: An evaluation and practitioner's guide. Res Synth Methods. 2018 Dec;9(4):602–14.

16. Higgins JP. Revised Cochrane risk-of-bias tool for randomized trials (RoB 2). In RoB2 Development Group; 2019. Available from: https://sites.google.com/site/riskofbiastool/welcome/rob-2-0-tool/current-version-of-rob-2

17. The Nordic Cochrane Centre, The Cochrane Collaboration. Review Manager (RevMan). Copenhagen; 2014.

18. Inrig JK, Califf RM, Tasneem A, Vegunta RK, Molina C, Stanifer JW, et al. The Landscape of Clinical Trials in Nephrology: A Systematic Review of ClinicalTrials.gov. Am J Kidney Dis Off J Natl Kidney Found. 2014 May;63(5):771–80.

19. Speich B, von Niederhäusern B, Schur N, Hemkens LG, Fürst T, Bhatnagar N, et al. Systematic review on costs and resource use of randomized clinical trials shows a lack of transparent and comprehensive data. J Clin Epidemiol. 2018 Apr 1;96:1–11.

For peer review only

Tables

Table 1 – Search strategy for Medline (Pubmed)

dialysis[tiab]	AND	phase ii/iii[tiab]
OR		OR
peritoneal dialysis[tiab]		treatment switching[tiab]
OR		OR
hemodialysis[tiab]		biomarker adaptive[tiab]
OR		OR
hemodiafiltration[tiab]		biomarker adaptive design[tiab]
OR		OR
haemodiafiltration[tiab]		biomarker adjusted[tiab]
OR		OR
hemofiltration[tiab]		adaptive hypothesis[tiab]
OR		OR
haemofiltration		adaptive dose-finding[tiab]
OR		OR
extracorporeal blood cleansing[tiab]		pick-the winner[tiab]
OR		OR
haemodialysis[tiab]		drop-the-loser[tiab]
OR		OR
Renal Dialysis[mh]		sample size re-estimation[tiab]
OR		OR
Renal replacement[tiab]		re-estimations[tiab]
OR		OR
end stage kidney[tiab]		adaptive randomization[tiab]
OR		OR
end stage renal[tiab]		group sequential[tiab]
OR		OR
stage 5 kidney[tiab]		adaptive seamless[tiab]
OR		OR
stage 5 renal[tiab]		adaptive design[tiab]
		OR
		Interim monitoring[tiab]
		OR
		Bayesian adaptive[tiab]
		OR
		Flexible design[tiab]
		OR
		Adaptive trial[tiab]
		OR
		play-the-winner[tiab]
		OR
		adaptive method[tiab]
		OR
		(adaptive[All Fields] AND dose[All Fields] AND adjusting[All Fields])
		OR
		response adaptive[All Fields]
		OR
		adaptive allocation[All Fields]
		OR
		adaptive signature design[tiab]
		OR
		treatment adaptive[tiab]
		OR
		covariate adaptive[tiab]

For peer review only

OR
sample size adjustment[tiab].

Table 2 – Characteristics of the trials

Study Characteristic	Categories	Description
Nature of Adaptive Design	GSD/SSR/DS/DE/Seamless/Interim Analysis	The type of adaptive design used in the trial.
Stopping Rule	Futility/Efficacy/Two sided/ N/A	If a stopping rule was used, what was the nature of the stopping rule.
Year of study completion	None	The year of study completion.
Population under study	None	A description of the population studied e.g. patients with diabetes.
Chronicity of RRT	Acute Kidney Injury (AKI) / ESKD	A category for the chronity of Renal Replacement Therapy (RRT), either Acute Kidney Injury (AKI) or End Stage Renal Disease (ESRD).
Intervention	None	A free text description of the intervention.
Nature of the intervention	Medication/Medical Device/Dialysis Parameter	A category for the nature of the intervention.
Primary Outcome	None	A description of the primary outcome of the trial.
Type of primary outcome	Continuous or dichotomous	A categorial variable for the type of primary outcome variable.
Nature of primary outcome	Surrogate, patient-centered or hard clinical	A categorial variable for the nature of primary outcome variable either surrogate, patient-centered or hard clinical.
Dialysis Modality	Haemodialysis, peritoneal dialysis, haemodiafiltration or haemofiltration	A categorial variable for the dialysis modality.
Sample Size of Study	None	The number of participants in the study.
The country of the lead investigator.	None	The country of the lead investigator.
The funder of the study	Public/Private	A categorial variable for source of funding for the study.
Study Phase	Phase II/Phase III/Combined Phase II/III	A categorial variable for study phase.

For peer review only

PRISMA-P 2015 checklist: recommended items to address in a systematic review protocol*

Section and topic	Item No	Checklist item	Page
**ADMINISTRATIVE INFORMATION**			**Page**
Title:			
Identification	1a	Identify the report as a protocol of a systematic review	1
Update	1b	If the protocol is for an update of a previous systematic review, identify as such	N/A
Registration	2	If registered, provide the name of the registry (such as PROSPERO) and registration number	5
Authors:			
Contact	3a	Provide name, institutional affiliation, e-mail address of all protocol authors; provide physical mailing address of corresponding author	1
Contributions	3b	Describe contributions of protocol authors and identify the guarantor of the review	10
Amendments	4	If the protocol represents an amendment of a previously completed or published protocol, identify as such and list changes; otherwise, state plan for documenting important protocol amendments	N/A
Support:			
Sources	5a	Indicate sources of financial or other support for the review	10
Sponsor	5b	Provide name for the review funder and/or sponsor	10
Role of sponsor or funder	5c	Describe roles of funder(s), sponsor(s), and/or institution(s), if any, in developing the protocol	10
**INTRODUCTION**			
Rationale	6	Describe the rationale for the review in the context of what is already known	4
Objectives	7	Provide an explicit statement of the question(s) the review will address with reference to participants, interventions, comparators, and outcomes (PICO)	4
**METHODS**			
Eligibility criteria	8	Specify the study characteristics (such as PICO, study design, setting, time frame) and report characteristics (such as years considered, language, publication status) to be used as criteria for eligibility for the review	5

For peer review only

Information sources	9	Describe all intended information sources (such as electronic databases, contact with study authors, trial registers or other grey literature sources) with planned dates of coverage	5
Search strategy	10	Present draft of search strategy to be used for at least one electronic database, including planned limits, such that it could be repeated	6, 13
Study records:			
Data management	11a	Describe the mechanism(s) that will be used to manage records and data throughout the review	5
Selection process	11b	State the process that will be used for selecting studies (such as two independent reviewers) through each phase of the review (that is, screening, eligibility and inclusion in meta-analysis)	7
Data collection process	11c	Describe planned method of extracting data from reports (such as piloting forms, done independently, in duplicate), any processes for obtaining and confirming data from investigators	7
Data items	12	List and define all variables for which data will be sought (such as PICO items, funding sources), any pre-planned data assumptions and simplifications	7, 14
Outcomes and prioritization	13	List and define all outcomes for which data will be sought, including prioritization of main and additional outcomes, with rationale	7, 14
Risk of bias in individual studies	14	Describe anticipated methods for assessing risk of bias of individual studies, including whether this will be done at the outcome or study level, or both; state how this information will be used in data synthesis	8
Data synthesis	15a	Describe criteria under which study data will be quantitatively synthesised	8
	15b	If data are appropriate for quantitative synthesis, describe planned summary measures, methods of handling data and methods of combining data from studies, including any planned exploration of consistency (such as I^2, Kendall's τ)	N/A
	15c	Describe any proposed additional analyses (such as sensitivity or subgroup analyses, meta-regression)	8
	15d	If quantitative synthesis is not appropriate, describe the type of summary planned	8
Meta-bias(es)	16	Specify any planned assessment of meta-bias(es) (such as publication bias across studies, selective reporting within studies)	N/A
Confidence in cumulative evidence	17	Describe how the strength of the body of evidence will be assessed (such as GRADE)	8

*** It is strongly recommended that this checklist be read in conjunction with the PRISMA-P Explanation and Elaboration (cite when available) for important clarification on the items. Amendments to a review protocol should be tracked and dated. The copyright for PRISMA-P (including checklist) is held by the PRISMA-P Group and is distributed under a Creative Commons Attribution Licence 4.0.**

For peer review only

From: Shamseer L, Moher D, Clarke M, Ghersi D, Liberati A, Petticrew M, Shekelle P, Stewart L, PRISMA-P Group. Preferred reporting items for systematic review and meta-analysis protocols (PRISMA-P) 2015: elaboration and explanation. BMJ. 2015 Jan 2;349(jan02 1):g7647.

For peer review only

PRISMA-P 2015 checklist: recommended items to address in a systematic review protocol*

Section and topic	Item No	Checklist item	Page
**ADMINISTRATIVE INFORMATION**			**Page**
Title:			
Identification	1a	Identify the report as a protocol of a systematic review	1
Update	1b	If the protocol is for an update of a previous systematic review, identify as such	N/A
Registration	2	If registered, provide the name of the registry (such as PROSPERO) and registration number	5
Authors:			
Contact	3a	Provide name, institutional affiliation, e-mail address of all protocol authors; provide physical mailing address of corresponding author	1
Contributions	3b	Describe contributions of protocol authors and identify the guarantor of the review	10
Amendments	4	If the protocol represents an amendment of a previously completed or published protocol, identify as such and list changes; otherwise, state plan for documenting important protocol amendments	N/A
Support:			
Sources	5a	Indicate sources of financial or other support for the review	10
Sponsor	5b	Provide name for the review funder and/or sponsor	10
Role of sponsor or funder	5c	Describe roles of funder(s), sponsor(s), and/or institution(s), if any, in developing the protocol	10
**INTRODUCTION**			
Rationale	6	Describe the rationale for the review in the context of what is already known	4
Objectives	7	Provide an explicit statement of the question(s) the review will address with reference to participants, interventions, comparators, and outcomes (PICO)	4
**METHODS**			
Eligibility criteria	8	Specify the study characteristics (such as PICO, study design, setting, time frame) and report characteristics (such as years considered, language, publication status) to be used as criteria for eligibility for the review	5

For peer review only

Information sources	9	Describe all intended information sources (such as electronic databases, contact with study authors, trial registers or other grey literature sources) with planned dates of coverage	5
Search strategy	10	Present draft of search strategy to be used for at least one electronic database, including planned limits, such that it could be repeated	6, 13
Study records:			
Data management	11a	Describe the mechanism(s) that will be used to manage records and data throughout the review	5
Selection process	11b	State the process that will be used for selecting studies (such as two independent reviewers) through each phase of the review (that is, screening, eligibility and inclusion in meta-analysis)	7
Data collection process	11c	Describe planned method of extracting data from reports (such as piloting forms, done independently, in duplicate), any processes for obtaining and confirming data from investigators	7
Data items	12	List and define all variables for which data will be sought (such as PICO items, funding sources), any pre-planned data assumptions and simplifications	7, 14
Outcomes and prioritization	13	List and define all outcomes for which data will be sought, including prioritization of main and additional outcomes, with rationale	7, 14
Risk of bias in individual studies	14	Describe anticipated methods for assessing risk of bias of individual studies, including whether this will be done at the outcome or study level, or both; state how this information will be used in data synthesis	8
Data synthesis	15a	Describe criteria under which study data will be quantitatively synthesised	8
	15b	If data are appropriate for quantitative synthesis, describe planned summary measures, methods of handling data and methods of combining data from studies, including any planned exploration of consistency (such as I^2, Kendall's τ)	N/A
	15c	Describe any proposed additional analyses (such as sensitivity or subgroup analyses, meta-regression)	8
	15d	If quantitative synthesis is not appropriate, describe the type of summary planned	8
Meta-bias(es)	16	Specify any planned assessment of meta-bias(es) (such as publication bias across studies, selective reporting within studies)	N/A
Confidence in cumulative evidence	17	Describe how the strength of the body of evidence will be assessed (such as GRADE)	8

*** It is strongly recommended that this checklist be read in conjunction with the PRISMA-P Explanation and Elaboration (cite when available) for important clarification on the items. Amendments to a review protocol should be tracked and dated.** **The copyright for PRISMA-P (including checklist) is held by the PRISMA-P Group and is distributed under a Creative Commons Attribution Licence 4.0.**

For peer review only

From: Shamseer L, Moher D, Clarke M, Ghersi D, Liberati A, Petticrew M, Shekelle P, Stewart L, PRISMA-P Group. Preferred reporting items for systematic review and meta-analysis protocols (PRISMA-P) 2015: elaboration and explanation. BMJ. 2015 Jan 2;349(jan02 1):g7647.

For peer review only

BMJ Open

Adaptive design methods in dialysis clinical trials– a systematic review protocol

Journal:	*BMJ Open*
Manuscript ID	bmjopen-2019-036755.R1
Article Type:	Protocol
Date Submitted by the Author:	25-Apr-2020
Complete List of Authors:	Judge, Conor; National University of Ireland Galway, Department of Medicine<br>Murphy, Robert P; National University of Ireland Galway, ; University Hospital Galway,<br>Cormican, Sarah; National University of Ireland Galway, Department of Medicine<br>Smyth, Andrew; National University of Ireland Galway, Department of Medicine<br>O'Halloran, Martin; National University of Ireland Galway, Department of Medicine<br>O'Donnell, Martin; National University of Ireland Galway, Department of Medicine
**Primary Subject Heading**:	Research methods
Secondary Subject Heading:	Renal medicine, Research methods
Keywords:	Dialysis < NEPHROLOGY, End stage renal failure < NEPHROLOGY, Clinical trials < THERAPEUTICS

SCHOLARONE™ Manuscripts

I, the Submitting Author has the right to grant and does grant on behalf of all authors of the Work (as defined in the below author licence), an exclusive licence and/or a non-exclusive licence for contributions from authors who are: i) UK Crown employees; ii) where BMJ has agreed a CC-BY licence shall apply, and/or iii) in accordance with the terms applicable for US Federal Government officers or employees acting as part of their official duties; on a worldwide, perpetual, irrevocable, royalty-free basis to BMJ Publishing Group Ltd ("BMJ") its licensees and where the relevant Journal is co-owned by BMJ to the co-owners of the Journal, to publish the Work in this journal and any other BMJ products and to exploit all rights, as set out in our licence.

The Submitting Author accepts and understands that any supply made under these terms is made by BMJ to the Submitting Author unless you are acting as an employee on behalf of your employer or a postgraduate student of an affiliated institution which is paying any applicable article publishing charge ("APC") for Open Access articles. Where the Submitting Author wishes to make the Work available on an Open Access basis (and intends to pay the relevant APC), the terms of reuse of such Open Access shall be governed by a Creative Commons licence – details of these licences and which Creative Commons licence will apply to this Work are set out in our licence referred to above.

Other than as permitted in any relevant BMJ Author's Self Archiving Policies, I confirm this Work has not been accepted for publication elsewhere, is not being considered for publication elsewhere and does not duplicate material already published. I confirm all authors consent to publication of this Work and authorise the granting of this licence.

For peer review only

Title

Adaptive design methods in dialysis clinical trials– a systematic review protocol

Authors

Conor Judge, M.B., BEng. (1) (2) (3)

Robert Murphy, M.B. (1)

Sarah Cormican, M.B. (1)

Andrew Smyth, M.B., Ph.D. (1)

Martin O Halloran, BEng., Ph.D. (2)

Martin J. O'Donnell, M.B., Ph.D. (1)

Author affiliations

(1) HRB-Clinical Research Facility, NUI Galway, Galway, Ireland

(2) Translational Medical Device Lab, NUI Galway, Galway, Ireland

(3) Wellcome Trust – HRB, Irish Clinical Academic Training

Corresponding Author

Dr Conor Judge, HRB-Clinical Research Facility, National University of Ireland Galway, Newcastle Road, Galway, Ireland, H91YR71 Email: conorjudge@gmail.com

For peer review only

Word Count

1893

For peer review only

Abstract

Introduction

Adaptive design methods are a potential solution to improve efficiency of clinical trials but their uptake in dialysis is unknown. We aim to investigate the use of adaptive design methods in dialysis clinical trials and to cultivate further adoption of adaptive trials by the nephrology community.

Methods and analysis

We will adhere to the Preferred Reporting Items for Systematic Review and Meta-Analysis Protocols (PRISMA-P) guidelines and the Cochrane Collaboration Handbook. We will perform a literature search though MEDLINE (Pubmed), EMBASE and CENTRAL, a detailed data extraction of trial characteristics and a narrative synthesis of the data. There will be no language restrictions. We will estimate the percentage of adaptive clinical trials per year in dialysis. Sub-group analysis will be performed by dialysis modality, funder and geographical location.

Ethics and dissemination

Ethical approval will not be required for this study as data will be obtained from publicly available clinical trials. We will disseminate our results in a peer-reviewed publication.

Registration Number

PROSPERO: 163946 (Temporary ID)

Keywords

Dialysis, Adaptive design, Haemodialysis, Peritoneal Dialysis, End Stage Renal Disease

For peer review only

Article Summary

Strengths and Limitations

- This study presents a comprehensive methodology for a systematic review of the current state of adaptive designs (AD) clinical trials in dialysis.
- Two researchers will independently perform the study selection, data extraction and risk of bias assessment.
- Detailed characteristics of trials will be collected and analysed including nature of intervention, funding source, dialysis modality, and the nature of the adaptive design.
- Subgroup analysis will be performed to highlight differences in design characteristics between dialysis modality, temporal trends, types of intervention, funding and geographical location.

For peer review only

Introduction

Background

Randomised controlled trials are the gold standard for confirming efficacy or futility of new therapies (1). Nephrology as a speciality, and especially patients with End Stage Renal Disease (ESRD), has traditionally had a low number of randomised trials compared to similar specialities (2). Many reasons have been cited for this including recruitment difficulty, history of underpowered trials and lack of funding (3,4). Nephrology studies are increasing in number but still lag behind other specialities (5).

There are an estimated 726,331 prevalent cases of ESRD in the United States (6) and globally, in 2010, an estimated 2.3–7.1 million people with end-stage kidney disease died without access to chronic dialysis (7). Projections estimate a 11%–18% increase in the crude incidence rate from 2015 to 2030 (8). People with ESRD represent 1% of the Medicare population but account for 7% of Medicare's expenditures (9). Additionally, people with End Stage Renal Disease experience higher mortality (10), morbidity and worse quality of life than the general public (11).

Adaptive clinical trials use the results from interim data analysis of ongoing trials to modify the study design in a predefined way (12). This is performed without undermining the integrity or validity of the trial and thus preserving the Type 1 Error (False Positive) rate. The most common type of adaptive design is the Group Sequential Trial, where planned interim analysis enables stopping of trials for efficacy or futility. Other designs included sample size re-estimation, multi-arm multi-stage trials, adaptive randomisation, biomarker adaptive and seamless phase II/III trials (13). Adaptive clinical trials would appear particularly suitable for evaluation of novel interventions in ESRD and according to the Food and Drug Administration, adaptive designs could reduce resource requirements, decrease time to study completion and increase the likelihood of study success (14). A specific problem in previous ESRD trials includes over reliance on observational data in the design of

For peer review only

clinical trials (15). Observational data are used to inform assumptions about the expected effect size and standard deviation of effect size for power calculations. If these assumptions are incorrect, they can result in an underpowered trial with an insufficient sample size to answer the research question. Adaptive sample size re-estimation has been used successfully in cardiology trials to help this problem (16). Planned blinded sample size re-estimation can discover incorrect underlying assumptions and trigger increased recruitment targets mid trial to ensure adequate power to answer the research question. A second specific problem in previous ESRD trials is extrapolation of results from non-CKD populations. An example of this was the 4D study (17), where 20mg/day of Atorvastatin in patients with diabetes and ESRD did not reduce cardiovascular events, despite showing a 20-30% reduction in non-CKD populations (18). One concern was that a single level of statin or a single low-density lipoprotein cut-off would not convey the same benefit in a ESKD population compared to a non-CKD population (15). Adaptive multi-arm multi-stage (MAMS) trials have been successfully used in HIV trials, Telmisartan and Insulin Resistance in HIV (TAILoR) used a multi-arm multi-stage design with one interim analysis to access three telmisartan doses (20, 40 and 80mg daily) (19). MAMS designs are partially suited for in ESKD populations where, compared to non-CKD populations, pharmacokinetic differences can be large and alternative drug doses are often required (20).

Objectives

The systematic review will aim to: 1) summarise the use of adaptive design methods in dialysis clinical trials per year, 2) describe the characteristics of the adaptive designs including dialysis modality, funding, and geographical location, 3) describe the adaptive design characteristics of the trial, 4) estimate the percentage of adaptive clinical trials in dialysis and 5) outline trends in all the above.

For peer review only

Methods and analysis

This systematic review has been registered with PROSPERO (registration number: 163946 (Temporary ID)). We will conduct this systematic review according to the Preferred Reporting Items for Systematic review and Meta-analysis Protocols (PRISMA-P) and have completed the PRISMA-P checklist (Supplementary Appendix 1).

Inclusion/exclusion criteria for the selection of studies

Type of study design and participants

Randomised controlled trials of interventions in patients with End Stage Renal Disease or acute kidney injury undergoing renal replacement therapy including haemodialysis, peritoneal dialysis, haemodiafiltration and haemofiltration. We will not limit our population or any specific disease.

Type of intervention

We will not place a restriction on the intervention type and will include trials that study medications during dialysis, medical devices, dialysis parameters etc.

Type of outcome

We will include all outcomes including surrogate markers, patient-centred outcomes and hard clinical outcomes.

Search method for the identification of trials

Electronic search

We will perform electronic searches on MEDLINE (Pubmed), EMBASE and CENTRAL from database inception until 01 January 2020. Zotero will be used as our reference manager and the Revtools

For peer review only

package on R will be used to eliminate duplicate records. The search will be conducted in English. The dialysis search terms were adapted from Beaubien-Souligny et al. 2019 (21) and include dialysis[tiab] OR peritoneal dialysis[tiab] OR hemodialysis[tiab] OR hemodiafiltration[tiab] OR haemodiafiltration[tiab] OR hemofiltration[tiab] OR haemofiltration OR extracorporeal blood cleansing[tiab] OR haemodialysis[tiab] OR Renal Dialysis[mh] OR Renal replacement[tiab] OR end stage kidney[tiab] OR end stage renal[tiab] OR stage 5 kidney[tiab] OR stage 5 renal[tiab]).

The adaptive design search terms were adapted from Bothwell et al., 2018 (22) and include "phase ii/iii"[tiab] OR "treatment switching"[tiab] OR "biomarker adaptive"[tiab] OR "biomarker adaptive design"[tiab] OR "biomarker adjusted"[tiab] OR "adaptive hypothesis"[tiab] OR "adaptive dose-finding"[tiab] OR "pick-thewinner"[tiab] OR "drop-the-loser"[tiab] OR "sample size re-estimation"[tiab] OR "re-estimations"[tiab] OR "adaptive randomization"[tiab] OR "group sequential"[tiab] OR "adaptive seamless"[tiab] OR "adaptive design"[tiab] OR "Interim monitoring"[tiab] OR "Bayesian adaptive"[tiab] OR "Flexible design"[tiab] OR "Adaptive trial"[tiab] OR "play-the-winner"[tiab] OR "adaptive method"[tiab] OR (adaptive[All Fields] AND dose[All Fields] AND adjusting[All Fields]) OR "response adaptive"[All Fields] OR "adaptive allocation"[All Fields] OR "adaptive signature design"[tiab] OR "treatment adaptive"[tiab] OR "covariate adaptive"[tiab] OR "sample size adjustment"[tiab]. We will perform two searches, firstly, we will combine the dialysis and adaptive search terms using a Boolean AND. The specific search will be carried out as described in Table 1. Secondly, we will perform a search with the dialysis search terms without the adaptive search terms for calculation of our total randomised controlled trials in dialysis denominator.

Selection and analysis of trials

We will use the high sensitivity machine learning classifier (RobotSearch) to identify randomised controlled trials from the combined search (dialysis and adaptive design) and from the dialysis search (23). RobotSearch is a machine learning classification algorithm combining an ensemble of

For peer review only

Support Vector Machines (SVM) and Convolutional Neural Networks (CNN) with a reported Area Under the Curve of 0.987 (95% Confidence Interval (CI), 0.984 to 0.989). We will manually confirm a random sample (10%) of studies classified as “Not RCT” after the RobotSearch screening step. We will then review the title and abstracts of studies to confirm that they are RCTs and identify trials with adaptive design methods for inclusion or exclusion. Studies with insufficient information to determine use of adaptive design methods will also be included for full text review. We will then perform full text review to confirm studies that will be included in the final systematic review. This process will be summarised in a PRISMA flowchart. Abstract, title and full text review will be performed by CJ and RM. Disagreements will be resolved by consensus or by a third reviewer (SC), if necessary.

CJ and RM will extract the following information (Adapted from Hatfield et al. 2016) in parallel and record in a custom database (Summarised in Table 2):

- The type of the adaptive design e.g. dose-finding, adaptive hypothesis, group sequential, adaptive randomisation, seamless phase II/III, adaptive treatment-switching, biomarker adaptive, sample size re-estimation.
- Stopping rule (Futility or efficacy).
- Year of completion of study.
- Trial population.
- Duration of time participants were receiving renal replacement therapy (Acute Kidney Injury or End Stage Kidney Disease).
- Intervention.
- Domain of the intervention (Medication, medical device or dialysis parameter).
- Primary outcome measure.
- Type of primary outcome variable (Continuous or dichotomous).

For peer review only

- Nature of primary outcome (Surrogate outcomes, patient-reported measures or hard clinical outcomes).
- Dialysis Modality (haemodialysis, peritoneal dialysis, haemodiafiltration or haemofiltration).
- Sample Size.
- The country of the lead investigator.
- The funder of the study (Public or Private).
- Published in journal with Impact Factor >10.

Assessment of the quality of the studies: risk of bias

We will use the Cochrane Risk of Bias Tool (24) to assess methodological quality of eligible trials including random sequence generation, allocation concealment, blinding of participants and health care personnel, blinded outcome assessment, completeness of outcome data, evidence of selective reporting and other biases. Risk of bias assessments will be performed independently by two reviewers (SC, RM), and disagreements were resolved by a third reviewer (CJ). If two of the domains was rated as high, the study was considered at high risk of bias. We will create a risk of bias summary table using Review Manager 5.3 (25).

Data synthesis

A descriptive synthesis of the data will be performed. We will estimate the percentage of adaptive clinical trials in dialysis per year by dividing the adaptive clinical trials per year by the total number of randomised controlled trials per year.

Analysis by subgroups

We will report overall outcomes and outcomes by dialysis modality (haemodialysis, peritoneal dialysis, haemodiafiltration and haemofiltration).

For peer review only

Study status

This systematic review will start in April 2020.

Patient and public involvement

There was no formal patient and public involvement in the design of this study.

Ethics and dissemination

Ethical approval was not required for this study. We will publish the results of this systematic review in a peer reviewed journal.

Discussion

The number of clinical trials performed in End Stage Renal Disease is low compared to other medical subspecialties (26). Additionally, the cost of conducting randomised controlled trials are rising (27) and there are reduced funding sources available, especially for nephrology (4). Adaptive clinical trials hold the potential to increase the efficiency and number of randomized controlled trials in End Stage Renal Disease and dialysis.

We expect to provide the following results: first, we will report on the proportion of randomised controlled trials in dialysis that include an adaptive design, second, we will outline the most popular type of Adaptive design in End Stage Renal Disease trials, third, we will report the main dialysis modalities where adaptive designs are used and possibly highlight underutilisation in additional modalities and fourth, we will report the geographical locations and funding pattern for trials using adaptive design in dialysis.

Furthermore, the main impact from this systematic review will be increased awareness in the nephrology community of the potential benefit of using adaptive designs in clinical trials. Having

For peer review only

211 clear examples and use cases of successful adaptive design will stimulate further use of these designs. ESKD patients are often excluded from randomised controlled trials, we urgently need novel design methods for investigating treatments in this underserved population. This systematic review will highlight adaptive designs as one part of the solution to this problem. A secondary impact from this systematic review will be an up to date census of all randomised controlled trials in dialysis with a yearly count of trials. This information can be used to influence funders and policy makers about the importance of funding nephrology and especially ESKD research.

Limitations

This review will have potential limitations including publication and reporting bias. We will not be able to include studies with unpublished data and we will mis-classify studies that do not have clear reporting of adaptive designs in their methodology.

For peer review only

Contributors

CJ, RB and MOD designed the study. CJ, RB, SC, AS, MOH, MOD reviewed and approved the final version of the protocol. CJ is the guarantor of the review.

Funding

This work was performed within the Irish Clinical Academic Training (ICAT) Programme, supported by the Wellcome Trust and the Health Research Board (Grant Number 203930/B/16/Z), the Health Service Executive, National Doctors Training and Planning and the Health and Social Care, Research and Development Division, Northern Ireland. The funding source had no role in the study design, analysis or writing of report.

Competing interests

None Declared.

Patient consent for publication

Not required.

Provenance and peer review

For peer review only

References

1. Bothwell LE, Greene JA, Podolsky SH, Jones DS. Assessing the Gold Standard — Lessons from the History of RCTs. N Engl J Med. 2016 Jun 2;374(22):2175–81.

2. Kovesdy CP. Clinical trials in end-stage renal disease—priorities and challenges. Nephrol Dial Transplant. 2019 Jul 1;34(7):1084–9.

3. Baigent C, Herrington WG, Coresh J, Landray MJ, Levin A, Perkovic V, et al. Challenges in conducting clinical trials in nephrology: conclusions from a Kidney Disease—Improving Global Outcomes (KDIGO) Controversies Conference. Kidney Int. 2017 Aug 1;92(2):297–305.

4. Bryan L, Ibrahim T, Zent R, Fischer MJ. The Kidney Research Predicament. J Am Soc Nephrol JASN. 2014 May;25(5):898–903.

5. Chatzimanouil MKT, Wilkens L, Anders H-J. Quantity and Reporting Quality of Kidney Research. J Am Soc Nephrol. 2019 Jan 1;30(1):13–22.

6. UNITED STATES RENAL DATA SYSTEM: Chapter 1: Incidence, Prevalence, Patient Characteristics, and Treatment Modalities 2018. [Internet]. [cited 2019 Nov 18]. Available from: http://www.usrds.org

7. Liyanage T, Ninomiya T, Jha V, Neal B, Patrice HM, Okpechi I, et al. Worldwide access to treatment for end-stage kidney disease: a systematic review. The Lancet. 2015 May;385(9981):1975–82.

8. McCullough KP, Morgenstern H, Saran R, Herman WH, Robinson BM. Projecting ESRD Incidence and Prevalence in the United States through 2030. J Am Soc Nephrol. 2019 Jan 1;30(1):127–35.

9. UNITED STATES RENAL DATA SYSTEM: Chapter 9: Healthcare Expenditures for Persons with ESRD [Internet]. 2018 [cited 2019 Nov 18]. Available from: http://www.usrds.org

10. Robinson BM, Zhang J, Morgenstern H, Bradbury BD, Ng LJ, McCullough KP, et al. Worldwide, mortality risk is high soon after initiation of hemodialysis. Kidney Int. 2014 Jan 1;85(1):158–65.

11. Wyld M, Morton RL, Hayen A, Howard K, Webster AC. A Systematic Review and Meta-Analysis of Utility-Based Quality of Life in Chronic Kidney Disease Treatments. Turner N, editor. PLoS Med. 2012 Sep 11;9(9):e1001307.

12. Chow S-C, Chang M, Pong A. Statistical Consideration of Adaptive Methods in Clinical Development. J Biopharm Stat. 2005 Jul 1;15(4):575–91.

13. Pallmann P, Bedding AW, Choodari-Oskooei B, Dimairo M, Flight L, Hampson LV, et al. Adaptive designs in clinical trials: why use them, and how to run and report them. BMC Med [Internet]. 2018 Dec [cited 2020 Apr 23];16(1). Available from: https://bmcmedicine.biomedcentral.com/articles/10.1186/s12916-018-1017-7

14. Research C for DE and. Adaptive Design Clinical Trials for Drugs and Biologics [Internet]. U.S. Food and Drug Administration. 2019 [cited 2019 Nov 18]. Available from: http://www.fda.gov/regulatory-information/search-fda-guidance-documents/adaptive-design-clinical-trials-drugs-and-biologics

For peer review only

15. Novak JE, Inrig JK, Patel UD, Califf RM, Szczech LA. Negative trials in nephrology: what can we learn? Kidney Int. 2008 Nov;74(9):1121–7.

16. Chaitman BR. Effects of Ranolazine With Atenolol, Amlodipine, or Diltiazem on Exercise Tolerance and Angina Frequency in Patients With Severe Chronic AnginaA Randomized Controlled Trial. JAMA. 2004 Jan 21;291(3):309.

17. Wanner C, Krane V, März W, Olschewski M, Mann JFE, Ruf G, et al. Atorvastatin in Patients with Type 2 Diabetes Mellitus Undergoing Hemodialysis. N Engl J Med. 2005 Jul 21;353(3):238–48.

18. Cheung BMY, Lauder IJ, Lau C-P, Kumana CR. Meta-analysis of large randomized controlled trials to evaluate the impact of statins on cardiovascular outcomes. Br J Clin Pharmacol. 2004 May;57(5):640–51.

19. Pushpakom S, Kolamunnage-Dona R, Taylor C, Foster T, Spowart C, García-Fiñana M, et al. TAILoR (TelmisArtan and InsuLin Resistance in Human Immunodeficiency Virus [HIV]): An Adaptive-design, Dose-ranging Phase IIb Randomized Trial of Telmisartan for the Reduction of Insulin Resistance in HIV-positive Individuals on Combination Antiretroviral Therapy. Clin Infect Dis [Internet]. 2019 Jul 3 [cited 2020 Apr 23]; Available from: https://academic.oup.com/cid/advance-article/doi/10.1093/cid/ciz589/5527878

20. Velenosi TJ, Urquhart BL. Pharmacokinetic considerations in chronic kidney disease and patients requiring dialysis. Expert Opin Drug Metab Toxicol. 2014 Aug;10(8):1131–43.

21. Beaubien-Souligny W, Kontar L, Blum D, Bouchard J, Denault AY, Wald R. Meta-analysis of Randomized Controlled Trials Using Tool-Assisted Target Weight Adjustments in Chronic Dialysis Patients. Kidney Int Rep. 2019 Jul;S2468024919314044.

22. Bothwell LE, Avorn J, Khan NF, Kesselheim AS. Adaptive design clinical trials: a review of the literature and ClinicalTrials.gov. BMJ Open. 2018 Feb 1;8(2):e018320.

23. Marshall IJ, Noel-Storr A, Kuiper J, Thomas J, Wallace BC. Machine learning for identifying Randomized Controlled Trials: An evaluation and practitioner's guide. Res Synth Methods. 2018 Dec;9(4):602–14.

24. Higgins JP. Revised Cochrane risk-of-bias tool for randomized trials (RoB 2). In RoB2 Development Group; 2019. Available from: https://sites.google.com/site/riskofbiastool/welcome/rob-2-0-tool/current-version-of-rob-2

25. The Nordic Cochrane Centre, The Cochrane Collaboration. Review Manager (RevMan). Copenhagen; 2014.

26. Inrig JK, Califf RM, Tasneem A, Vegunta RK, Molina C, Stanifer JW, et al. The Landscape of Clinical Trials in Nephrology: A Systematic Review of ClinicalTrials.gov. Am J Kidney Dis Off J Natl Kidney Found. 2014 May;63(5):771–80.

27. Speich B, von Niederhäusern B, Schur N, Hemkens LG, Fürst T, Bhatnagar N, et al. Systematic review on costs and resource use of randomized clinical trials shows a lack of transparent and comprehensive data. J Clin Epidemiol. 2018 Apr 1;96:1–11.

For peer review only

Tables

Table 1 – Search strategy for Medline (Pubmed)

dialysis[tiab]	AND	phase ii/iii[tiab]
OR		OR
peritoneal dialysis[tiab]		treatment switching[tiab]
OR		OR
hemodialysis[tiab]		biomarker adaptive[tiab]
OR		OR
hemodiafiltration[tiab]		biomarker adaptive design[tiab]
OR		OR
haemodiafiltration[tiab]		biomarker adjusted[tiab]
OR		OR
hemofiltration[tiab]		adaptive hypothesis[tiab]
OR		OR
haemofiltration		adaptive dose-finding[tiab]
OR		OR
extracorporeal blood cleansing[tiab]		pick-the winner[tiab]
OR		OR
haemodialysis[tiab]		drop-the-loser[tiab]
OR		OR
Renal Dialysis[mh]		sample size re-estimation[tiab]
OR		OR
Renal replacement[tiab]		re-estimations[tiab]
OR		OR
end stage kidney[tiab]		adaptive randomization[tiab]
OR		OR
end stage renal[tiab]		group sequential[tiab]
OR		OR
stage 5 kidney[tiab]		adaptive seamless[tiab]
OR		OR
stage 5 renal[tiab]		adaptive design[tiab]
		OR
		Interim monitoring[tiab]
		OR
		Bayesian adaptive[tiab]
		OR
		Flexible design[tiab]
		OR
		Adaptive trial[tiab]
		OR
		play-the-winner[tiab]
		OR
		adaptive method[tiab]
		OR
		(adaptive[All Fields] AND dose[All Fields] AND adjusting[All Fields])
		OR
		response adaptive[All Fields]
		OR
		adaptive allocation[All Fields]
		OR
		adaptive signature design[tiab]
		OR
		treatment adaptive[tiab]
		OR
		covariate adaptive[tiab]

For peer review only

	OR<br>sample size adjustment[tiab].

Table 2 – Characteristics of the trials

Study Characteristic	Categories	Description
Nature of Adaptive Design	GSD/SSR/DS/DE/Seamless/Interim Analysis	The type of adaptive design used in the trial.
Stopping Rule	Futility/Efficacy/Two sided/ N/A	If a stopping rule was used, what was the nature of the stopping rule.
Year of study completion	None	The year of study completion.
Population under study	None	A description of the population studied e.g. patients with diabetes.
Chronicity of RRT	Acute Kidney Injury (AKI) / ESKD	A category for the chronity of Renal Replacement Therapy (RRT), either Acute Kidney Injury (AKI) or End Stage Renal Disease (ESRD).
Intervention	None	A free text description of the intervention.
Nature of the intervention	Medication/Medical Device/Dialysis Parameter	A category for the nature of the intervention.
Primary Outcome	None	A description of the primary outcome of the trial.
Type of primary outcome	Continuous or dichotomous	A categorial variable for the type of primary outcome variable.
Nature of primary outcome	Surrogate, patient-centered or hard clinical	A categorial variable for the nature of primary outcome variable either surrogate, patient-centered or hard clinical.
Dialysis Modality	Haemodialysis, peritoneal dialysis, haemodiafiltration or haemofiltration	A categorial variable for the dialysis modality.
Sample Size of Study	None	The number of participants in the study.
The country of the lead investigator.	None	The country of the lead investigator.
The funder of the study	Public/Private	A categorial variable for source of funding for the study.
Study Phase	Phase II/Phase III/Combined Phase II/III	A categorial variable for study phase.

For peer review only

For peer review only

PRISMA-P 2015 checklist: recommended items to address in a systematic review protocol*

Section and topic	Item No	Checklist item	
**ADMINISTRATIVE INFORMATION**			**Page**
Title:			
Identification	1a	Identify the report as a protocol of a systematic review	1
Update	1b	If the protocol is for an update of a previous systematic review, identify as such	N/A
Registration	2	If registered, provide the name of the registry (such as PROSPERO) and registration number	5
Authors:			
Contact	3a	Provide name, institutional affiliation, e-mail address of all protocol authors; provide physical mailing address of corresponding author	1
Contributions	3b	Describe contributions of protocol authors and identify the guarantor of the review	12
Amendments	4	If the protocol represents an amendment of a previously completed or published protocol, identify as such and list changes; otherwise, state plan for documenting important protocol amendments	N/A
Support:			
Sources	5a	Indicate sources of financial or other support for the review	12
Sponsor	5b	Provide name for the review funder and/or sponsor	12
Role of sponsor or funder	5c	Describe roles of funder(s), sponsor(s), and/or institution(s), if any, in developing the protocol	12
**INTRODUCTION**			
Rationale	6	Describe the rationale for the review in the context of what is already known	4
Objectives	7	Provide an explicit statement of the question(s) the review will address with reference to participants, interventions, comparators, and outcomes (PICO)	4
**METHODS**			
Eligibility criteria	8	Specify the study characteristics (such as PICO, study design, setting, time frame) and report characteristics (such as years considered, language, publication status) to be used as criteria for eligibility for the review	6

For peer review only

Information sources	9	Describe all intended information sources (such as electronic databases, contact with study authors, trial registers or other grey literature sources) with planned dates of coverage	6
Search strategy	10	Present draft of search strategy to be used for at least one electronic database, including planned limits, such that it could be repeated	6, 17
Study records:			
Data management	11a	Describe the mechanism(s) that will be used to manage records and data throughout the review	7
Selection process	11b	State the process that will be used for selecting studies (such as two independent reviewers) through each phase of the review (that is, screening, eligibility and inclusion in meta-analysis)	8
Data collection process	11c	Describe planned method of extracting data from reports (such as piloting forms, done independently, in duplicate), any processes for obtaining and confirming data from investigators	8
Data items	12	List and define all variables for which data will be sought (such as PICO items, funding sources), any pre-planned data assumptions and simplifications	8, 18
Outcomes and prioritization	13	List and define all outcomes for which data will be sought, including prioritization of main and additional outcomes, with rationale	8, 18
Risk of bias in individual studies	14	Describe anticipated methods for assessing risk of bias of individual studies, including whether this will be done at the outcome or study level, or both; state how this information will be used in data synthesis	9
Data synthesis	15a	Describe criteria under which study data will be quantitatively synthesised	9
	15b	If data are appropriate for quantitative synthesis, describe planned summary measures, methods of handling data and methods of combining data from studies, including any planned exploration of consistency (such as I^2, Kendall's τ)	N/A
	15c	Describe any proposed additional analyses (such as sensitivity or subgroup analyses, meta-regression)	9
	15d	If quantitative synthesis is not appropriate, describe the type of summary planned	9
Meta-bias(es)	16	Specify any planned assessment of meta-bias(es) (such as publication bias across studies, selective reporting within studies)	N/A
Confidence in cumulative evidence	17	Describe how the strength of the body of evidence will be assessed (such as GRADE)	9

*** It is strongly recommended that this checklist be read in conjunction with the PRISMA-P Explanation and Elaboration (cite when available) for important clarification on the items. Amendments to a review protocol should be tracked and dated.** **The copyright for PRISMA-P (including checklist) is held by the PRISMA-P Group and is distributed under a Creative Commons Attribution Licence 4.0.**

From: Shamseer L, Moher D, Clarke M, Ghersi D, Liberati A, Petticrew M, Shekelle P, Stewart L, PRISMA-P Group. Preferred reporting items for systematic review and meta-analysis protocols (PRISMA-P) 2015: elaboration and explanation. BMJ. 2015 Jan 2;349(jan02 1):g7647.

For peer review only
